# Erythrocyte Transfusion as an Independent Predictor of Survival but Not Immune-Related Toxicity in Nivolumab-Treated Metastatic NSCLC: A Multicenter Cohort

**DOI:** 10.3390/medicina62081601

**Published:** 2026-08-20

**Authors:** Mehmet Cem Fidan, Ebru Çiçek, Hamza Abbasov, Murad Guliyev, Emir Çerme, Kübra Akkaya, Bekir Doğan, Nargiz Majidova, Burak Paçacı, Ezgi Türkoğlu, Merve Ekinci Fidan, Emir Çelik, Mesut Yılmaz, İbrahim Vedat Bayoğlu, Özkan Alan, Nebi Serkan Demirci

**Affiliations:** 1 Division of Medical Oncology, Department of Internal Medicine, Cerrahpasa Faculty of Medicine, Istanbul University-Cerrahpasa, Istanbul 34098, Türkiye; 2 Department of Medical Oncology, Prof. Dr. Cemil Tascıoglu City Hospital, University of Health Sciences, Istanbul 34360, Türkiye; 3 Department of Medical Oncology, Bakirkoy Dr. Sadi Konuk Training and Research Hospital, University of Health Sciences, Istanbul 34147, Türkiye; 4 Department of Medical Oncology, VM Medical Park Maltepe Hospital, Istanbul 34846, Türkiye; 5 Division of Medical Oncology, Department of Internal Medicine, School of Medicine, Marmara University, Istanbul 34899, Türkiye; 6 Department of Medical Oncology, Kartal Dr. Lutfi Kirdar City Hospital, Health Science University, Istanbul 34865, Türkiye; 7 Department of Thoracic Surgery, Yedikule Chest Diseases and Thoracic Surgery Education and Research Hospital, Istanbul 34025, Türkiye; merveekincifidan@gmail.com; 8 Department of Medical Oncology, Faculty of Medicine, Topkapi Liv Hospital, Istinye University, Istanbul 34010, Türkiye

**Keywords:** non-small cell lung cancer, nivolumab, blood transfusion, transfusion-related immunomodulation, immunotherapy, survival

## Abstract

*Background and Objectives*: Transfusion-related immunomodulation (TRIM) is a recognized immunosuppressive phenomenon whose interaction with immune checkpoint blockade remains poorly defined. We evaluated the impact of erythrocyte suspension transfusion on survival and immune-related toxicity in patients with metastatic non-small cell lung cancer (NSCLC) treated with nivolumab. *Materials and Methods*: This retrospective, multicenter study included 253 patients with metastatic NSCLC treated with nivolumab across five centers between April 2018 and March 2025. Patients who received a transfusion within three months before or during nivolumab therapy were compared with non-transfused patients. Survival was assessed using Kaplan–Meier and Cox regression analyses. *Results*: Forty-two patients (16.6%) received transfusion. Transfused patients had significantly shorter progression-free survival (PFS) (median 3.6 vs. 9.3 months; HR = 2.28, 95% CI 1.47–3.56, *p* < 0.001) and overall survival (OS) (median 5.8 vs. 16.3 months; HR = 2.76, 95% CI 1.86–4.11, *p* < 0.001). In multivariable analysis, transfusion was an independent predictor of both shorter PFS (HR = 2.288, 95% CI 1.470–3.561, *p* < 0.001) and shorter OS (HR = 2.351, 95% CI 1.565–3.533, *p* < 0.001), independent of transfusion volume. No statistically significant association was detected between transfusion status and the incidence of nivolumab-related toxicity (*p* = 0.585), although this analysis was likely underpowered, whereas nivolumab-related toxicity was an independent favorable prognostic factor. *Conclusions*: Erythrocyte transfusion independently predicted poorer survival but not immune-related toxicity in nivolumab-treated metastatic NSCLC, supporting more judicious transfusion practices during immunotherapy.

## 1. Introduction

According to the GLOBOCAN 2022 data, lung cancer is the second most common cancer worldwide and the leading cause of cancer-related death [1]. Five-year survival rates in metastatic-stage non-small cell lung cancer (NSCLC), which constitutes approximately 85% of lung cancers, are below 10% and continue to be a clinical problem [2].

Immune checkpoint inhibitors (ICIs) enhance the tumor-directed T-cell response by targeting the programmed cell death protein-1 (PD-1)/PD-L1 and cytotoxic T-lymphocyte-associated protein 4 (CTLA-4) axes, leading to a radical paradigm shift in cancer treatment [3]. Immunotherapy is widely used in oncology for solid organ malignancies at all stages. Nivolumab, which has proven its superiority over docetaxel in the second-line treatment of patients with metastatic NSCLC in the CheckMate-017 and CheckMate-057 studies, is one such indication [4,5]. Unfortunately, the desired clinical benefit is not observed in the vast majority of patients due to primary refractory disease or secondary resistance, highlighting the need for a better understanding of the medications and factors that can be administered concurrently or modified, and that affect the efficacy of immunotherapy in cancer patients [6,7,8,9,10,11,12].

Anemia, a common complication in patients with cancer, occurs due to multiple factors, such as tumor-related chronic disease, bone marrow infiltration, and myelosuppression due to chemotherapy. In some cases, erythrocyte suspension (ES) transfusion is required [13,14]. Transfusion-related immunomodulation (TRIM) was first described following the observation that pretransplant blood transfusions improved renal allograft survival in the late 1970s [15,16]. Various studies have shown that this immunomodulatory effect has clinically significant immunosuppressive effects [17]. The immunosuppressive consequences of TRIM include impaired natural killer (NK) cell cytotoxicity, reduced cluster of differentiation 8 (CD8+) cytotoxic T lymphocytes activity, and expansion of regulatory T (Treg) cells, collectively shifting the immune balance toward tolerance [18,19,20,21]. Importantly, the immunosuppressive effect of TRIM may be transient and is likely to be most clinically significant in the period surrounding the initiation of immunotherapy [12,22]. These mechanisms are specific to ES transfusion and differ from those of other transfusions; the effects of other transfusions on tumor cells and their interactions with ICI are not yet clinically understood [23,24].

The negative impact of blood transfusions on cancer prognosis was first demonstrated in animal studies. In 1981, Francis et al. experimentally showed that tumor growth increased in rats who received allogeneic blood transfusions [25,26]. In the clinical field, it was first reported in 1982 that perioperative ES transfusion increased the risk of tumor recurrence in patients with colorectal cancer [27], and this relationship was further strengthened by subsequent comprehensive meta-analyses [28]. Similar adverse associations have been reported for many different solid tumors, including lung, glioblastoma, ovarian, and head and neck cancers [29,30,31,32,33].

The first critical evidence on the intersection of TRIM and immunotherapy efficacy came from a retrospective study by Mispelbaum et al. in patients with various solid tumors [12]. In a retrospective study of patients with NSCLC treated with immunotherapy, ES transfusion was independently associated with shorter survival [34]. Although these studies are pioneering, their heterogeneous patient populations and small sample sizes necessitate further research.

In parallel, oncology practice has increasingly adopted Patient Blood Management principles, favoring restrictive transfusion thresholds (typically 7–8 g/dL), correction of the underlying cause of anemia, and minimization of allogeneic exposure. Whether these restrictive strategies carry additional relevance during immune checkpoint blockade, where TRIM could theoretically blunt efficacy, remains undefined, providing the rationale for the present study [35].

Therefore, our study included only patients with metastatic NSCLC who received nivolumab and underwent ES transfusion to ensure patient homogeneity and aimed to evaluate and better understand the clinical effects of transfusion on immunotherapy.

## 2. Materials and Methods

### 2.1. Study Design

This retrospective, multicenter study was conducted in five centers in Türkiye. Medical records of patients diagnosed with NSCLC and treated with nivolumab were reviewed. Data were collected, including baseline demographic and clinical characteristics, treatment history, pre- and post-nivolumab transfusion status, nivolumab-related toxicity, and survival outcomes.

### 2.2. Patient Population

Patients with a confirmed diagnosis of NSCLC who were older than 18 years, had received at least one course of nivolumab, and had received a blood transfusion three months before or during nivolumab treatment were included in the study. Patients with known epidermal growth factor receptor (EGFR), anaplastic lymphoma kinase (ALK), or ROS proto-oncogene 1 (ROS1) mutations and those with incomplete or insufficient clinical data were excluded from the study.

### 2.3. Efficacy Results

Overall survival (OS) and progression-free survival (PFS) were calculated according to blood transfusion status. OS was defined as the time from initiation of nivolumab treatment to death from any cause or to the last follow-up. PFS was defined as the time from initiation of nivolumab treatment to disease progression or death from any cause as assessed by the treating physician.

### 2.4. Ethical Considerations

The study was conducted in accordance with the principles of the Declaration of Helsinki and was approved by the relevant ethics committees; full approval details are provided in the Institutional Review Board Statement. The requirement for informed consent was waived due to the retrospective nature of the study.

### 2.5. Statistical Analysis

Statistical analyses were performed using IBM SPSS Statistics version 27.0 (IBM Corp., Armonk, NY, USA). Categorical variables were expressed as frequencies and percentages and compared between groups using the Chi-square test or Fisher’s exact test as appropriate. Continuous variables were expressed as median (range) and compared using the Mann–Whitney U test. The random distribution of missing data between groups was tested, and appropriate statistical analyses were applied.

Survival curves were estimated using the Kaplan–Meier method and compared using the log-rank test. Univariate Cox proportional hazards regression analysis was performed to evaluate variables that could affect OS and PFS. Variables with *p* < 0.20 in the univariate analysis were included in the multivariate Cox regression model to identify independent prognostic factors. Candidate covariates for the multivariable models comprised the clinical and demographic variables available for the entire cohort (age, sex, ECOG performance status, disease stage, metastatic sites, and PD-L1 status); systemic inflammatory and nutritional markers were not available and could not be included. Median follow-up was estimated using the reverse Kaplan–Meier method. The proportional hazards assumption was assessed using log-minus-log survival plots (Supplementary Figure S1) and a time-dependent covariate (transfusion × time) test; no significant violation was detected (*p* = 0.90 for OS, *p* = 0.47 for PFS). In the multivariate analysis, a two-sided *p*-value <0.05 was considered statistically significant.

## 3. Results

### 3.1. Patient Characteristics

A total of 253 patients with NSCLC who received nivolumab therapy between April 2018 and March 2025 were included in the study. The median age was 63 years (range, 34–81 years), and the majority of patients were male (76.3%) and current or former smokers (86.6%). Adenocarcinoma was the most common histological subtype (54.5%), followed by squamous cell carcinoma (36.8%). At the time of nivolumab initiation, 59.3% of the patients had de novo metastatic disease. Most patients had an Eastern Cooperative Oncology Group (ECOG) performance status of 0–1 (88.5%). Nivolumab was administered as second-line therapy in 80.6% of patients. The details are provided in Tables S1–S3.

### 3.2. Transfusion-Related Characteristics

Of the 253 patients, 42 (16.6%) received blood transfusions before or during nivolumab therapy. Among the transfused patients, 71.4% received a single unit, and 28.6% received two or more units. Pre-transfusion hemoglobin levels were ≥8 g/dL in 47.6% of the patients. A total of 61.9% of transfusions were performed within three months before the initiation of nivolumab treatment, and 38.1% were performed within the first three months of nivolumab treatment. The transfusion characteristics are summarized in Table 1. The median timing of transfusion relative to nivolumab initiation was 0.15 months (range −3.0 to 3.1). The median pre-transfusion hemoglobin was 7.8 g/dL (IQR 7.0–8.0). Transfusions were administered for symptomatic or progressive anemia in accordance with institutional practice.

### 3.3. Nivolumab-Related Toxicity

Nivolumab-related toxicity was observed in 37 (14.6%) patients. The most frequent toxicity was hypothyroidism (43.2%), followed by pneumonia (24.3%) and skin toxicity (13.5%), respectively. Only 5.5% and 3.6% of patients interrupted or permanently discontinued nivolumab treatment due to toxicities, the vast majority of which were grade 2 (59.5%). The toxicity characteristics are detailed in Table S4.

### 3.4. Baseline Characteristics by Transfusion Status

There were no statistically significant differences between the groups in terms of age, smoking status, smoking history, PD-L1 expression status, disease stage at diagnosis, or ECOG performance status. The transfusion group had a significantly higher proportion of female patients (35.7% vs. 21.3%, *p* = 0.045) and a higher proportion of patients with ≥2 metastatic sites (52.4% vs. 31.8%, *p* = 0.011). Data regarding smoking status were missing for 15 patients (5.9%), and the remaining 238 patients were included in the analysis. Data on PD-L1 expression were missing for 99 patients (39.1%), and the chi-square test indicated that this variable was randomly distributed across groups (*p* = 0.588). Therefore, PD-L1 analysis was performed on 154 patients with available data (Table 2).

### 3.5. Survival Outcomes

**Figure 1:**
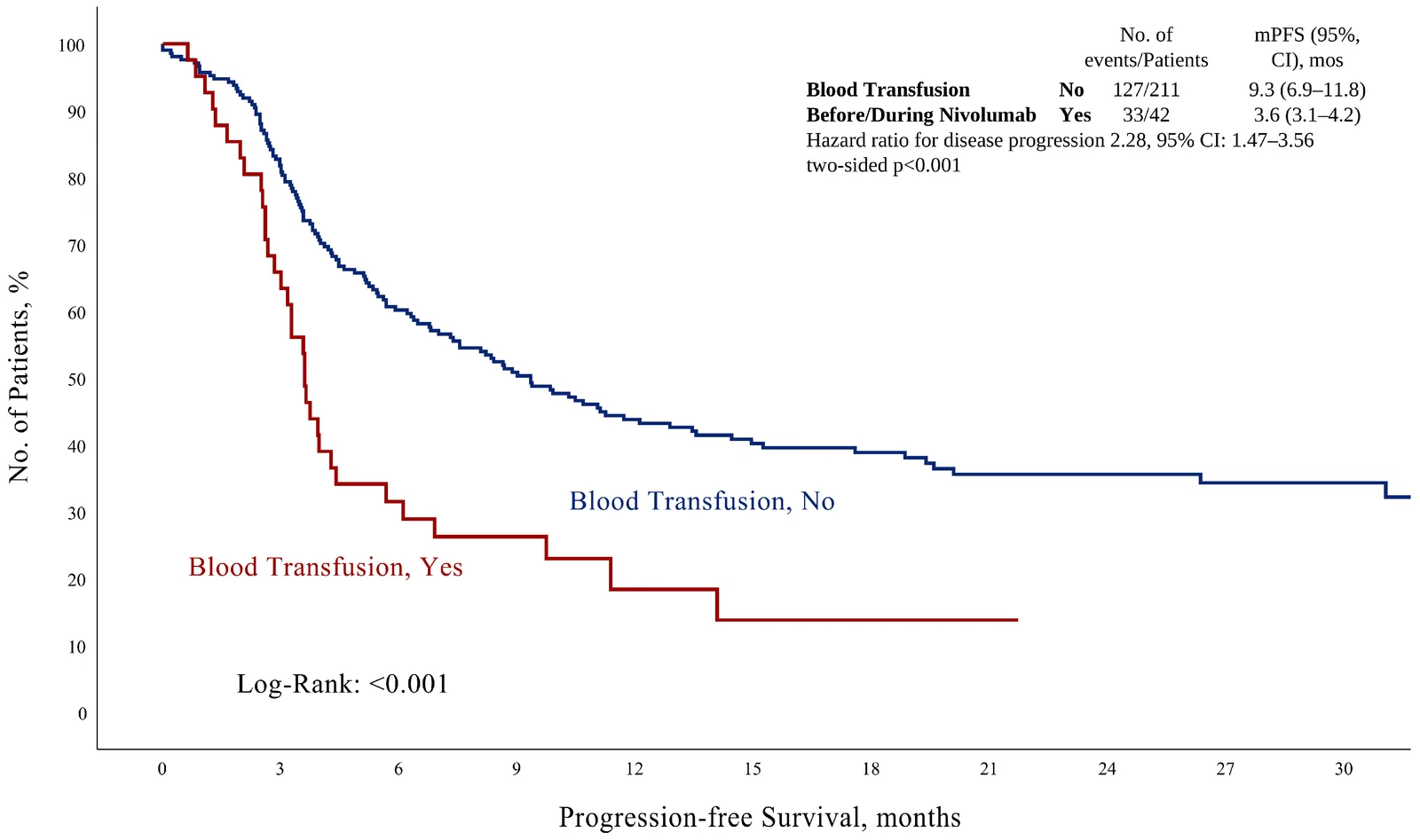
Kaplan–Meier Curves for Progression-Free Survival According to Blood Transfusion Status Before or During Nivolumab Therapy.

**Figure 2:**
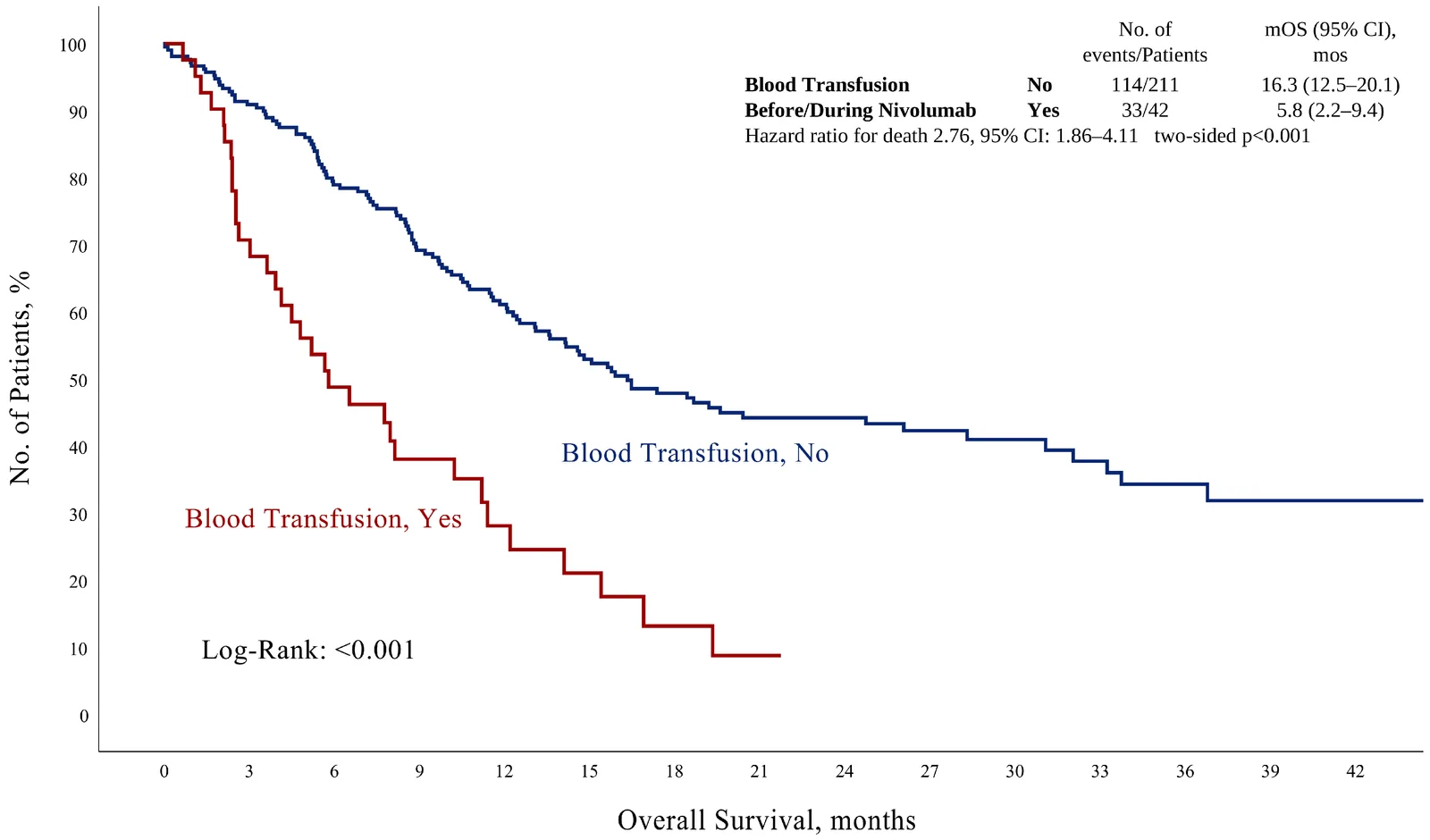
Kaplan–Meier Curves for Overall Survival According to Blood Transfusion Status Before or During Nivolumab Therapy.

At a median follow-up of 24.2 months, 147 of 253 patients (58.1%) had died, and 106 patients were censored. The number of events and patients per group is shown in Figure 1 and Figure 2.

Patients who received blood transfusions had significantly shorter PFS compared to those who did not receive transfusions; the median PFS was 3.6 months (95% confidence interval (CI): 3.1–4.2) and 9.3 months (95% CI: 6.9–11.8), respectively (Log-rank *p* < 0.001; hazard ratio (HR) = 2.28, 95% CI: 1.47–3.56) (Figure 1). Similarly, OS was also significantly shorter in the transfusion group; the median OS was 5.8 months (95% CI: 2.2–9.4), compared to 16.3 months (95% CI: 12.5–20.1) in the non-transfusion group (log-rank *p* < 0.001; HR = 2.76, 95% CI: 1.86–4.11) (Figure 2).

In a sensitivity analysis restricting the transfusion exposure to a ±60-day window around nivolumab initiation (35 transfused patients within this window), the association with shorter survival was preserved (OS HR = 2.85, 95% CI 1.87–4.34; PFS HR = 2.33, 95% CI 1.55–3.51). To address potential immortal time bias from transfusions administered after nivolumab initiation, a 60-day landmark analysis was also performed and yielded consistent results (OS HR = 3.04, 95% CI 1.97–4.68; PFS HR = 2.16, 95% CI 1.40–3.34).

### 3.6. Prognostic Factors for PFS

In the univariate analysis, age, female sex, newly developed metastatic disease at diagnosis, bone metastases, and blood transfusion were significantly associated with shorter PFS. In contrast, the presence of nivolumab-related toxicity was associated with better PFS. In the multivariate analysis, blood transfusion was the strongest independent predictor of shorter PFS (HR = 2.288, 95% CI: 1.470–3.561, *p* < 0.001), whereas nivolumab-related toxicity retained its favorable prognostic effect (HR = 0.559, 95% CI: 0.329–0.948, *p* = 0.031) (Table 3).

### 3.7. Prognostic Factors for OS

In the univariate analysis, female sex, ECOG ≥ 2, pleural effusion/nodules, bone metastases, and blood transfusion before or during nivolumab administration were significantly associated with OS. In the univariate analysis, blood transfusion was the strongest predictor of shorter OS (HR = 2.763, 95% CI: 1.857–4.111, *p* < 0.001), whereas nivolumab-related toxicity was associated with improved OS (HR = 0.532, 95% CI: 0.311–0.918, *p* = 0.021). Pre-transfusion hemoglobin was excluded from the multivariable models because it was defined only in transfused patients and has no equivalent value in non-transfused patients, precluding its use as a cohort-wide covariate. In the multivariable analysis, blood transfusion remained an independent predictor of shorter OS (HR = 2.351, 95% CI: 1.565–3.533, *p* < 0.001), together with female sex (HR = 0.635, *p* = 0.017), ECOG ≥ 2 (HR = 1.731, *p* = 0.032), pleural effusion/nodules (HR = 1.833, *p* = 0.022), and bone metastases (HR = 1.512, *p* = 0.022); nivolumab-related toxicity retained its favorable prognostic effect (HR = 0.529, 95% CI: 0.307–0.910, *p* = 0.021) (Table 4).

## 4. Discussion

In this multicenter retrospective study of patients with metastatic NSCLC receiving nivolumab and erythrocyte suspension (ES) transfusion, blood transfusion was an independent predictor of both shorter PFS and shorter OS in multivariable analysis. On the other hand, nivolumab-related toxicity emerged as an independent protective factor for both PFS and OS.

Anemia is a common complication in cancer patients and also negatively impacts survival [36,37]. Therefore, it is necessary to consider whether the factor affecting survival is anemia or the transfusion itself. The presence of anemia requiring transfusion (especially in the case of disease-related anemia) may indicate a more severe illness. To address this question, we recorded pre-transfusion hemoglobin levels; approximately half of the transfused patients (47.6%) had Hb ≥ 8 g/dL, while the remainder had more pronounced anemia. We therefore cannot attribute the survival difference to transfusion alone, as anemia severity may also contribute. Nevertheless, transfusion remained an independent predictor of poorer survival after multivariable adjustment, suggesting an effect beyond anemia itself.

On the other hand, in the transfused group, a statistically significantly higher proportion of patients with ≥2 metastatic sites was found (*p* = 0.011), which may indicate a higher tumor burden. This difference could have affected survival outcomes, but when the number of metastatic sites was also included in the model in multivariate analysis, the negative impact of transfusion on both PFS and OS continued independently.

First described in the 1970s [15,16], the immunological mechanisms of TRIM, which describe the immunosuppression state that develops after transfusion [18,19,20,21], can directly weaken the antitumor response targeted by immune checkpoint inhibitors. The association between transfusion and both shorter PFS and OS in our study is consistent with this pathophysiological basis, although a causal role of TRIM cannot be established from observational data.

While TRIM provides a biologically plausible framework, the observed association is unlikely to be attributable to a single mechanism. Transfusion requirement may itself serve as a surrogate marker of greater disease burden, systemic inflammation, and cancer cachexia, each independently linked to poorer immunotherapy outcomes. Because systemic inflammatory and nutritional markers (NLR, CRP, albumin, LDH) were not available cohort-wide, these competing explanations could not be fully excluded, and the association should be regarded as hypothesis-generating rather than as evidence of a direct causal effect.

It is known that the immunosuppressive effect of TRIM is temporary rather than permanent [12,22]. Therefore, the timing of the transfusion is particularly important. For this reason, in our study, we included transfusions performed in the first 3 months before and after the start of nivolumab. Thus, we aimed to evaluate our results during the period when the effect of TRIM-induced immunosuppression may still be present at the start of immunotherapy. Our results support the hypothesis that the effect of TRIM on immunotherapy efficacy is most evident at the time of treatment initiation [12,22]. However, it was found that whether the transfusion was performed before or during the treatment period did not significantly affect survival. This finding suggests that, within the three-month window studied, the precise timing of transfusion relative to nivolumab initiation did not differentially affect survival. Sensitivity analyses using a narrower ±60-day window and a 60-day landmark approach yielded consistent results, supporting the robustness of this association. In our study, transfusion volume did not significantly affect PFS or OS. This finding suggests that the negative impact of TRIM-induced immunosuppression, and thus on survival, is dependent on immunosuppression itself rather than the transfusion dose. For this reason, it is necessary to carefully review the transfusion indications in patients receiving immunotherapy.

In the literature, the negative effect of ES transfusion on survival in patients with solid tumors was first demonstrated by Mispelbaum et al. [12]. Our study supports these data with a homogeneous patient population and a larger sample size.

Another finding in our study was that the toxicity rate did not differ significantly between the transfused and non-transfused groups (*p* = 0.585). While it is a reasonable and logical expectation that there would be less toxicity in the transfused group given the nature of TRIM, no statistically significant association was detected; however, with only five toxicity events in the transfused group, this analysis was likely underpowered, and a clinically meaningful effect cannot be excluded. Many studies and meta-analyses show that the development of immunotherapy-related adverse events (irAEs) indicates an immunologically active response and is associated with improved survival [38,39]. In our study, it was also found that the survival times of patients experiencing nivolumab-related toxicity were statistically longer compared to those who did not experience toxicity. These findings are consistent with the literature.

In our study, data on PD-L1 expression status were missing in 39.1% of patients, which we acknowledge as a limitation. This likely reflects the treatment landscape during the study period, when immune checkpoint inhibitors were used predominantly in the second-line setting and beyond; as nivolumab does not require PD-L1-based patient selection in this setting, routine PD-L1 testing may not have been considered clinically necessary or cost-effective by treating physicians, which plausibly explains the missingness and is consistent with its random distribution across groups (*p* = 0.588). Importantly, nivolumab is approved for second-line NSCLC irrespective of PD-L1 expression, having demonstrated a survival benefit over docetaxel without PD-L1-based selection in the pivotal CheckMate-017 and CheckMate-057 trials [4,5]; the transfusion–survival association that is the focus of our study is therefore unlikely to be affected by the completeness of PD-L1 data. Consistent with this, PD-L1 was not associated with survival in our univariate analysis, although this is interpreted cautiously given the proportion of missing data. In this context, the aforementioned data deficiency is not expected to significantly affect the main findings of our study. Similarly, data on EGFR, ALK, and ROS1 mutations were also limited in our study; however, this deficiency was largely due to the squamous cell carcinoma subgroup, where the incidence of driver mutations is known to be quite low. In the CheckMate 057 study, EGFR and ALK mutations were detected in approximately 10% of patients [5]. However, next-generation sequencing (NGS) data for somatic mutations associated with primary resistance to immunotherapy, such as STK11/LKB1, KEAP1, and SMARCA4, were largely unavailable in our study [40,41,42]. The inclusion of patients carrying these mutations in the study may have adversely affected survival outcomes independently of transfusion, which constitutes a significant limitation of our study.

It is known that liver and brain metastases negatively affect the response to immunotherapy [43,44]. In our study, neither metastasis location nor survival showed a significant effect. This finding may be due to the relatively small number of patients in our study, and larger sample sizes may clarify the issue.

### Limitations

First, our study has potential limitations due to its retrospective design. Second, transfusion indications were not standardized, raising the possibility of confounding by indication: patients requiring transfusion may have had greater frailty, tumor burden, or disease severity not fully captured by the measured covariates, so residual confounding cannot be excluded despite multivariable adjustment. As a substantial proportion of transfused patients had pre-transfusion hemoglobin <8 g/dL, the contribution of anemia severity to the observed survival difference cannot be fully excluded. Third, because a proportion of transfusions were administered after nivolumab initiation, immortal time bias may have influenced the survival estimates; however, a 60-day landmark analysis addressing this bias yielded consistent results. Fourth, we did not apply propensity score-based methods (e.g., matching or inverse-probability weighting), which could have further improved covariate balance between groups. Fifth, key inflammatory and nutritional biomarkers (NLR, CRP, albumin, and LDH) were not available across the cohort and could not be retrospectively retrieved given the multicenter design, limiting our ability to adjust for systemic inflammation and cachexia as competing explanations. Finally, data on STK11/LKB1, KEAP1, and SMARCA4 mutations, known to be associated with primary resistance to immunotherapy, were missing, and their inclusion could have affected survival independently of transfusion. Despite these limitations, the multicenter structure of our study and its evaluation only on patients diagnosed with metastatic NSCLC and receiving nivolumab, the inclusion of transfusions performed before and only within a three-month timeframe considering the transient effect of TRIM, and the recording of pre-transfusion hemoglobin values, which allowed us to partially assess the contribution of anemia, are strengths of our study. Finally, in our study, we also evaluated the effect of transfusion status on nivolumab-related toxicity and found no significant difference in toxicity rates between the groups. This indicates that TRIM does not significantly affect immunotherapy-related adverse events; to our knowledge, this is the first data in this area.

## 5. Conclusions

This multicenter retrospective study reveals that erythrocyte transfusion has a significantly negative impact on both PFS and OS in metastatic NSCLC patients treated with nivolumab. Transfusion was identified as the strongest independent predictor of short PFS in multivariate analysis. Although the existence of this relationship, independent of transfusion volume, has not yet been confirmed, the underlying mechanism (possibly TRIM-mediated immunosuppression) suggests that even a single unit of transfusion may be sufficient to influence outcomes. After multivariable adjustment, transfusion remained an independent predictor of poorer survival, suggesting that the observed survival difference is not attributable to anemia severity alone. These findings highlight the importance of limiting the indications for erythrocyte transfusion in patients receiving immunotherapy. Designing a randomized controlled trial in patients requiring transfusion is not ethically feasible; depriving a patient with symptomatic anemia or an indication for transfusion by including them in the control group is incompatible with basic medical ethics. Therefore, evidence on this subject will necessarily be obtained from observational studies. Confirming our findings with larger sample sizes and prospective observational designs is important for advancing our understanding in this field.

## Figures and Tables

**Table 1:** Transfusion-Related Characteristics (*n* = 253).

		*n*	%
Blood Transfusion Before/During Nivolumab	No	211	83.4
	Yes	42	16.6
Number of Transfusion Units (transfused patients only, *n* = 42)	1	30	71.4
	≥2	12	28.6
Hemoglobin Level Prior to Transfusion (*n* = 42)	≥8 g/dL	20	47.6
	<8 g/dL	22	52.4
Timing of Transfusion Relative to Nivolumab (*n* = 42)	During nivolumab therapy (within 3 months after initiation)	16	38.1
	Within 3 months before nivolumab initiation	26	61.9

**Table 2:** Baseline Characteristics by Blood Transfusion Status in Nivolumab-Treated Patients (*n* = 253).

		Blood Transfusion Before/During Nivolumab				*p*	
		No (*n* = 211)		Yes (*n* = 42)			
**Age, years**	Median (min.–max.)	63 (31–84)		64 (51–80)		0.321	^m^
**Age, years**	<65	119	56.4	21	50.0	0.446	x^2^
	≥65	92	43.6	21	50.0		
**Sex, *n* (%)**	Female	45	21.3	15	35.7	***0.045***	x^2^
	Male	166	78.7	27	64.3		
**Smoking Status, *n* (%)**	No	15	7.5	4	10.8	0.490	x^2^
	Yes	186	92.5	33	89.2		
**Smoking History, *n* (%)**	Current	96	51.6	21	63.6	0.202	x^2^
	Former	90	48.4	12	36.4		
**PD-L1 Status, *n* (%)**	Negative	43	33.1	10	41.7	0.416	x^2^
	Positive	87	66.9	14	58.3		
**Disease Stage at Diagnosis, *n* (%)**	Early/Locally Advanced	85	40.3	18	42.9	0.757	x^2^
	De novo Metastatic	126	59.7	24	57.1		
**ECOG, *n* (%)**	0–1	187	88.6	36	85.7	0.594	x^2^
	≥2	24	11.4	6	14.3		
**Pleural Effusion/Nodule**	No	190	90.0	39	92.9	0.570	x^2^
	Yes	21	10.0	3	7.1		
**Bone**	No	156	73.9	25	59.5	0.059	x^2^
	Yes	55	26.1	17	40.5		
**Cranial**	No	174	82.5	33	78.6	0.550	x^2^
	Yes	37	17.5	9	21.4		
**Liver**	No	191	90.5	36	85.7	0.349	x^2^
	Yes	20	9.5	6	14.3		
**Number of Metastatic Sites**	0–1	144	68.2	20	47.6	***0.011***	x^2^
	≥2	67	31.8	22	52.4		

x^2^: Chi-square test, ^m^: Mann–Whitney U Test. Bold/italic values indicate statistical significance (*p* < 0.05).

**Table 3:** Univariate and Multivariate Cox Regression Analyses of Factors Associated with PFS.

	Univariate			Multivariate		
	HR	95%	*p*	HR	95%	*p*
**Age (<65 vs. ≥65)**	1.409	1.033–1.922	***0.030***			
**Sex (female vs. male)**	0.696	0.489–0.989	***0.043***			
**Smoking Status (no vs. yes)**	0.765	0.440–1.328	0.341			
**Smoking History (former vs. current)**	1.301	0.920–1.841	***0.136***			
**PD-L1 Status (positive vs. negative)**	1.275	0.826–1.969	0.273			
**Disease Stage at Diagnosis (locoregional vs. de novo metastatic)**	1.392	1.007–1.925	***0.045***			
**ECOG (0–1 vs. ≥2)**	1.480	0.926–2.368	***0.101***			
**Pleural Effusion/Nodule (no vs. yes)**	1.504	0.907–2.492	***0.114***			
**Bone Metastases (no vs. yes)**	1.453	1.041–2.027	***0.028***			
**Cranial Metastases (no vs. yes)**	0.818	0.533–1.253	0.355			
**Liver Metastases (no vs. yes)**	1.331	0.814–2.174	0.254			
**Number of Metastatic Sites (1 vs. ≥2)**	1.203	0.870–1.663	0.264			
**Blood Transfusion Before/During Nivolumab (no vs. yes)**	2.127	1.443–3.136	***<0.001***	2.288	1.470–3.561	***<0.001***
**Number of Transfusion Units (1 vs. ≥2)**	1.423	0.686–2.951	0.343			
**Timing of Transfusion (during/after vs. before)**	0.900	0.441–1.838	0.773			
**Nivolumab-Related Toxicity (no vs. yes)**	0.553	0.338–0.903	***0.018***	0.559	0.329–0.948	***0.031***

Bold/italic values indicate significance: *p* < 0.20 (univariate) and *p* < 0.05 (multivariable).

**Table 4:** Univariate and Multivariate Cox Regression Analyses of Factors Associated with OS.

	Univariate			Multivariate		
	HR	95%	*p*	HR	95%	*p*
**Age (<65 vs. ≥65)**	1.317	0.951–1.821	***0.097***			
**Sex (female vs. male)**	0.642	0.449–0.918	***0.015***	0.635	0.438–0.922	***0.017***
**Smoking Status (no vs. yes)**	0.811	0.464–1.417	0.461			
**Smoking History (former vs. current)**	1.249	0.862–1.809	0.239			
**PD-L1 Status (positive vs. negative)**	1.189	0.753–1.876	0.458			
**Disease Stage at Diagnosis (locoregional vs. de novo metastatic)**	1.404	0.998–1.975	***0.051***			
**ECOG (0–1 vs. ≥2)**	1.768	1.076–2.906	***0.024***	1.731	1.049–2.856	***0.032***
**Pleural Effusion/Nodule (no vs. yes)**	1.866	1.120–3.109	***0.018***	1.833	1.093–3.074	***0.022***
**Bone Metastases (no vs. yes)**	1.524	1.079–2.151	***0.017***	1.512	1.062–2.152	***0.022***
**Cranial Metastases (no vs. yes)**	0.970	0.626–1.504	0.893			
**Liver Metastases (no vs. yes)**	1.370	0.824–2.276	0.225			
**Number of Metastatic Sites (1 vs. ≥2)**	1.203	0.870–1.663	0.264			
**Blood Transfusion Before/During Nivolumab (no vs. yes)**	2.763	1.857–4.111	***<0.001***	2.351	1.565–3.533	***<0.001***
**Number of Transfusion Units (1 vs. ≥2)**	1.599	0.761–3.361	0.216			
**Timing of Transfusion (during/after vs. before)**	0.753	0.373–1.519	0.429			
**Nivolumab-Related Toxicity (no vs. yes)**	0.532	0.311–0.918	***0.021***	0.529	00.307–0.910	***0.021***

Bold/italic values indicate significance: *p* < 0.20 (univariate) and *p* < 0.05 (multivariable).

## Data Availability

The data presented in this study are available on request from the corresponding author.

## Supplementary Materials

The following supporting information can be downloaded at https://www.mdpi.com/article/10.3390/medicina62081601/s1. Table S1: Baseline Demographic and Clinical Characteristics of Patients (*n* = 253); Table S2: Treatment History (*n* = 253); Table S3: Metastatic Disease Characteristics at the Time of Nivolumab Initiation (*n* = 253); Table S4: Nivolumab-Related Toxicity (*n* = 253); Table S5: Summary of All Treatment Lines. Figure S1. log-minus-log survival plot for OS by transfusion status, with legend: Log-minus-log survival plot for overall survival by blood transfusion status, showing parallel, non-crossing curves consistent with the proportional hazards assumption.

## Abbreviations

ALK	Anaplastic lymphoma kinase
CD8+	Cluster of differentiation 8
CI	Confidence interval
CTLA-4	Cytotoxic T-lymphocyte-associated protein 4
ECOG	Eastern Cooperative Oncology Group
EGFR	Epidermal growth factor receptor
ES	Erythrocyte suspension
Hb	Hemoglobin
HR	Hazard ratio
ICI	Immune checkpoint inhibitor
irAE	Immunotherapy-related adverse event
NGS	Next-generation sequencing
NK	Natural killer
NSCLC	Non-small cell lung cancer
OS	Overall survival
PD-1	Programmed cell death protein-1
PD-L1	Programmed cell death ligand-1
PFS	Progression-free survival
ROS1	ROS proto-oncogene 1
TRIM	Transfusion-related immunomodulation
Treg	Regulatory T-cell
