# Supplementary material for: Erythrocyte Transfusion as an Independent Predictor of Survival but Not Immune-Related Toxicity in Nivolumab-Treated Metastatic NSCLC: A Multicenter Cohort
Source: Medicina (Kaunas). 2026 Aug 20;62(8):1601. doi: 10.3390/medicina62081601 (PMC13515754; doi:10.3390/medicina62081601)
Supplement: Supplementary file 1 [file medicina-62-01601-s001.zip › medicina-4459465-supplementary.pdf]

Supplementary Table S1. Baseline Demographic and Clinical Characteristics of Patients (n=253)

		n	%
**Age, years (median, min.max.)**		63, 34-81	
**Age**	<65	140	55,3
	≥65	113	44,7
**Sex**	Male	193	76,3
	Female	60	23,7
**Smoking Status**	No	19	7,5
	Yes	219	86,6
	Missing	15	5,9
**Smoking History**	Current	117	46,2
	Former	102	40,3
	Missing	34	13,4
**Pathology**	Adenocarcinoma	138	54,5
	Squamous Cell Carcinoma	93	36,8
	Adenosquamous Cell Carcinoma	5	2,0
	NOS	13	5,1
	Pleomorphic Carcinoma	3	1,2
	Squamous + Large Cell Carcinoma	1	0,4
**PD L1 status**	Negative	53	20,9
	Positive	101	39,9
	Missing	99	39,1
**EGR, ALK, ROS-1 status**	Negative	151	59,7
	Missing	102	40,3
**NGS Status**	Not performed	154	60,9
	Performed	96	37,9
	Missing	3	1,2
**Disease Stage at Diagnosis**	Early/Locally Advanced	103	40,7
	De novo Metastatic	150	59,3
**ECOG Performance Status at Nivolumab Initiation**	0-1	224	88,5
	≥2	29	11,5

NOS: Not otherwise specified

Supplementary Table S2. Treatment History (n=253)

		n	%
**Prior Surgery**	No	234	92,5
	Yes	17	6,7
**Treatment Received in Early/Locally Advanced Stage (n=103)**	Surgery → surveillance	2	1,9
	Surgery → adjuvant CT	15	14,6
	Neoadjuvant CT → surgery → adjuvant CT	2	1,9
	CRT → surgery → adjuvant CT	4	3,9
	Concurrent CRT	26	25,2
	Sequential CRT	21	20,4
	Definitive CRT → durvalumab maintenance	2	1,9
	Chemotherapy alone	9	8,7
	Radiotherapy alone	2	1,9
	Other	2	1,9
	Missing	18	17,5
**First-Line Chemotherapy Regimen**	Carboplatin + Gemcitabine	40	15,8
	Cisplatin + Gemcitabine	33	13,0
	Carboplatin + Vinorelbine	1	0,4
	Cisplatin + Vinorelbine	4	1,6
	Carboplatin + Pemetrexed	18	7,1
	Cisplatin + Pemetrexed	25	9,9
	Carboplatin + Paclitaxel	85	33,6
	Treatment in Early/Locally Advanced Stage	42	16,6
	Cisplatin + Etoposide	2	0,8
	Other	3	1,2
**Line of Nivolumab Therapy**	2nd line	204	80,6
	3rd line	35	13,8
	4th line	11	4,3
	5th line	3	1,2

Supplementary Table S3. Metastatic Disease Characteristics at the Time of Nivolumab Initiation (n=253)

		n	%
**Ipsilateral Lung Recurrence or Satellite Nodule**	No	203	80,2
	Yes	50	19,8
**Mediastinum**	No	132	52,2
	Yes	121	47,8
**Contralateral Lung**	No	205	81,0
	Yes	48	19,0
**Pleural Effusion/Nodule**	No	229	90,5
	Yes	24	9,5
**Bone**	No	181	71,5
	Yes	72	28,5
**Cranial**	No	207	81,8
	Yes	46	18,2
**Liver**	No	227	89,7
	Yes	26	10,3
**Adrenal**	No	227	89,7
	Yes	26	10,3
**Intra-abdominal Lymph Node**	No	246	97,2
	Yes	7	2,8
**Other**	No	231	91,3
	Yes	22	8,7
**Number of Metastatic Sites (Stage IV patients only, n=202)**	1	113	55,9
	2	58	28,7
	≥3	31	15,3

Supplementary Table S4. Nivolumab-Related Toxicity (n=253)

		n	%
**Nivolumab-Related Toxicity**	No	216	85,4
	Yes	37	14,6
**Type of Toxicity (n=37)**	Pneumonitis	9	24,3
	Cutaneous Toxicity	5	13,5
	Colitis	2	5,4
	Hypothyroidism	16	43,2
	Adrenal Insufficiency	2	5,4
	Polyneuropathy	2	5,4
	Cardiac Adverse Event	1	2,7
**Toxicity Grade (n=37)**	1	3	8,1
	2	22	59,5
	3	11	29,7
	5	1	2,7
**Nivolumab Interruption / Discontinuation Due to Toxicity**	No	230	90,9
	Interruption	14	5,5
	Permanent Discontinuation	9	3,6

Supplmentary Table S5. Summary of All Treatment Lines

		n	%
**Secon-Line Treatment**	Nivolumab	206	81,4
	Docetaxel	16	6,3
	Pemetrexed	8	3,2
	Gemcitabine	7	2,8
	Platin-Pemetrexed	4	1,6
	Platin-Gemcitabine	2	0,8
	Platin-Vinorelbine	3	1,2
	Carboplatin-Paklitaxel	1	0,4
	Other	6	2,4
**Third-Line Treatment**	Second-line treatment ongoing/not received	147	58,1
	Nivolumab	32	12,6
	Docetaxel	26	10,3
	Pemetrexed	8	3,2
	Gemcitabine	20	7,9
	Vinorelbine	7	2,8
	Platin-Pemetrexed	4	1,6
	Platin-Gemsitabine	3	1,2
	Platin-Vinorelbine	1	0,4

	Carboplatin-Paklitaxel	3	1,2
	Other	2	0,8
**Fourth-Line Treatment**	Third-line treatment ongoing/not received	207	81,8
	Nivolumab	13	5,1
	Docetaxel	4	1,6
	Pemetrexed	1	0,4
	Gemcitabine	11	4,3
	Vinorelbine	11	4,3
	Carboplatin-Paklitaxel	6	2,4

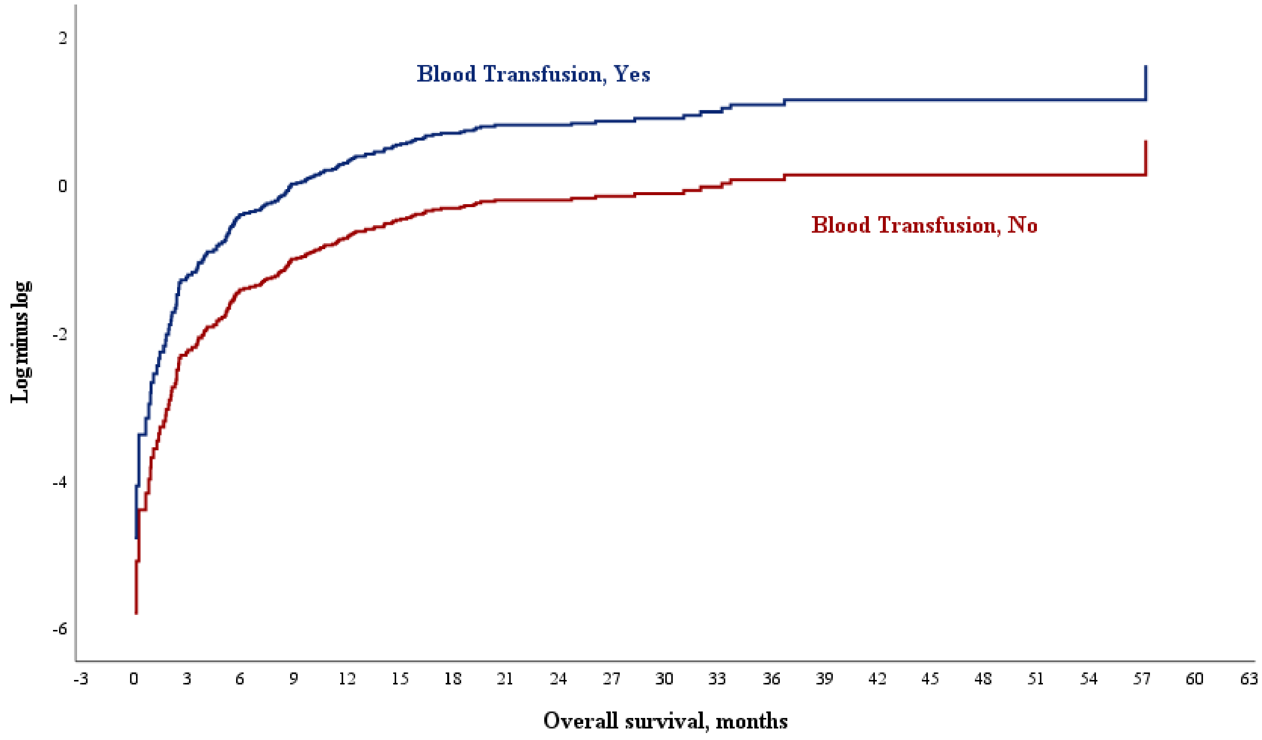

Supplementary Figure S1 — log-minus-log survival plot for OS by transfusion status, with legend: *Log-minus-log survival plot for overall survival by blood transfusion status, showing parallel, non-crossing curves consistent with the proportional hazards assumption.*
